# The relationship between toll like receptor 4 gene rs4986790 and rs4986791 polymorphisms and sepsis susceptibility: A meta-analysis

**DOI:** 10.1038/srep38947

**Published:** 2016-12-13

**Authors:** Rui Liu, Yuan-Yuan Mo, Hui-Li Wang, Yan Tan, Xiu-Jie Wen, Man-Jing Deng, Hong Yan, Lei Li

**Affiliations:** 1 Department of Stomatology, Daping Hospital and Research Institute of Surgery, Third Military Medical University, Chongqing 400038, People’s Republic of China; 2 Department of Infection and Immunity, State Key Laboratory of Trauma, Burn and Combined Injury, Daping Hospital and Research Institute of Surgery, Third Military Medical University, Chongqing 400038, People’s Republic of China; 3 Department of Nursing, Xi’an International University, Xi’an 710000, People’s Republic of China; 4 Department of Anesthesiology, Daping Hospital and Research Institute of Surgery, Third Military Medical University, Chongqing 400038, People’s Republic of China

## Abstract

Accumulating evidences have demonstrated that lipopolysaccharide (LPS) represents the important etiologic factor for sepsis. Some previous studies have reported the relationship between common polymorphisms rs4986790 and rs4986791 in the coding gene for this receptor and the susceptibility to sepsis, but there were distinct divergences between those findings. We therefore designed this meta-analysis incorporated 28 published articles containing 6,537 sepsis patients and 8,832 controls for a more comprehensive conclusion on this matter. Odds ratios (ORs) and 95% confidence interval (95% CIs) were calculated to evaluate the association of toll like receptor 4 gene polymorphisms rs4986790 and rs4986791 with sepsis risk. Heterogeneity between included studies was inspected using Q test, and sensitivity analysis was implemented via sequential deletion of each included study to investigate the stability of overall estimates. Funnel plot and Egger’s test were adopted to examine publication bias across selected studies. We found no significant association for either the polymorphism rs4986790 or rs4986791 with sepsis susceptibility in total analysis under any genetic models. Neither did we after combining these two polymorphisms. The results of this meta-analysis suggest that the rs4986790 and rs4986791 polymorphisms in toll like receptor 4 gene may have no statistically significant influence on sepsis susceptibility.

Sepsis is a systemic inflammatory response syndrome (SIRS) induced by organism infections from pathogenic microorganisms^1^. Organisms identify pathogen microorganisms through innate immunity system, thereby starting protective inflammatory response to eliminate pathogenic microorganisms, but over-response of systemic inflammation in organism may cause sepsis or septic shock^2^. Thousands of patients undergo target organ damage and multiple organ dysfunction caused by severe sepsis and septic shock annually, and the death rate of sepsis is still increasing constantly despite the development of medical condition and technology^3^. Although the precise etiology of sepsis remains unclear, relevant studies have shown that gene polymorphism plays important role in affecting individual susceptibility to sepsis^4^,^5^,^6^. Consequently, key gene polymorphisms involved in immune response pathways have been detected their influence on sepsis onset^7^,^8^.

Innate immune system depends on pattern recognition receptors (PRRs) to detect conserved structures of pathogenic microorganisms (like bacteria, virus, fungus and protozoa), which are called pathogen-associated molecular patterns (PAMPs)^9^. Toll like receptors (TLRs) play an important role in autoimmunity, and one of them, toll like receptor 4, occupies an extremely significant position as a receptor recognizing PAMPs^10^. Reportedly, polymorphisms in the gene coding for this receptor may change the extracellular structures of the protein and affect the combinations with pathogen ligands, especially the combination with lipopolysaccharide (LPS) of gram negative bacteria^11^, thereby changing the progression of sepsis. Studies have demonstrated that toll like receptor 4 gene polymorphism rs4986790 (also known as Asp299Gly or +896 A > G) increased the risk of severe sepsis^12^,^13^. In addition, Shalhub *et al*. indicated that apart from the polymorphism rs4986790, another one rs4986791 (also known as Thr399Ile) in this gene was also related to the severity of post-traumatic sepsis^14^. Whereas Jessen *et al*. showed that there were no obvious association between polymorphisms in the gene toll like receptor 4 and sepsis caused by gram negative bacteria in their study^15^. Feterowski *et al*. found that the morbidity and mortality rate of sepsis didn’t correlate with toll like receptor 4 gene mutations in their study on postoperative sepsis induced by various microorganism infections^16^.

It is important in terms of clinical value and theoretical significance to get clearer perspective on the relationship of toll like receptor 4 gene polymorphisms rs4986790 and rs4986791 with sepsis risk. Consequently, we systematically analyzed this correlation via the method of meta-analysis in this study.

## Materials and Methods

### Literature search and selection criteria

We searched potentially relevant studies from MEDLINE, EMBASE, Google Scholar and CNKI up to January 2016. The key terms adopted in search strategy included “toll like receptor 4 or *TLR4*”, “polymorphism or mutation or variant” and “sepsis”. What’s more, we also searched for additional articles through manually screening the reference lists of relevant articles or reviews to minimize the possibility of missing potent papers. Eligible studies were enrolled in this meta-analysis based on the following criteria: they estimated the correlation of the rs4986790 and/or rs4986791 polymorphisms in toll like receptor 4 gene with sepsis and had a case-control design; they adopted valid genotyping method; and they provided information about genotype distribution in case and control group for calculating pooled odds ratios (ORs) with the corresponding 95% confidence intervals (95% CIs). Meanwhile, publications were excluded if they were case-only studies, with irrelevant title or abstract, without sufficient genotype data or focusing on animals.

### Data extraction

The following information were extracted by two investigators from included articles: the first author’s name, publication year, original country, ethnicity, control source, genotyping method, numbers of cases and controls, genotype frequency and *P* values for Hardy-Weinberg equilibrium (HWE) in control group. All disagreements over extracted data were resolved through discussion between the two investigators to reach a consensus.

### Statistical analysis

The ORs and 95% CIs were calculated to assess the association of sepsis susceptibility with toll like receptor 4 rs4986790 polymorphism under five genetic models: GG vs. AA, GG + GA vs. AA, GG vs. GA+AA, allele G vs. allele A, and GA vs. GG, as well as with the polymorphism rs4986791 under the five genetic models: TT vs. CC, TT+CT vs. CC, TT vs. CC+CT, allele T vs. allele C and CT vs. CC. In addition, subgroup analysis was implemented according to ethnicity. Between-study heterogeneity was evaluated through the χ^2^-based Q test. The fixed-effect model was adopted to calculate the pooled ORs when *P* > 0.05 in Q test which indicated the lack of heterogeneity; otherwise, the random-effect model was selected. HWE was checked in the control group via χ^2^ test. Publication bias was examined with Begg’s funnel plot and Egger’s test. Sensitivity analysis was performed to reflect the effect of each individual dataset on pooled results through sequentially omitting each included study. Statistical analysis was conducted using STATA version 12.0 (STATA Corp). *P* < 0.05 was considered statistically significant for all tests.

## Results

### Characteristics of studies

**Figure 1:**
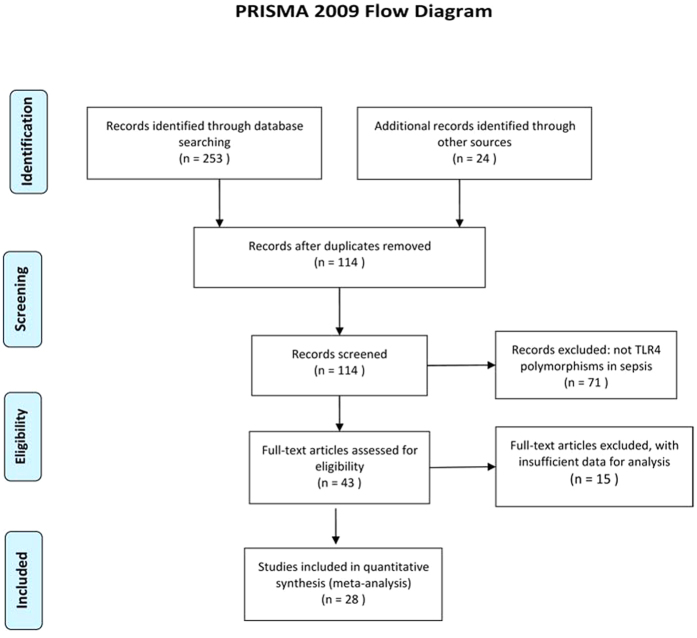
Flow diagram for study selection.

According to the search strategy, 277 potentially relevant studies were initially identified from MEDLINE, EMBASE, Google Scholar and CNKI, and 163 of them were exclude due to irrelevant titles or abstracts. Additionally, consequent screening removed 86 more reports for not concerning on the association between the polymorphism rs4986790 or rs4986791 of toll like receptor 4 and sepsis (71) and insufficient data (15). Therefore, as showed in Fig. 1, this meta-analysis finally included 28 eligible articles, containing 51 independent case-control studies (31 for the polymorphism rs4986790 and 20 for rs4986791)^13^,^14^,^16^,^17^,^18^,^19^,^20^,^21^,^22^,^23^,^24^,^25^,^26^,^27^,^28^,^29^,^30^,^31^,^32^,^33^,^34^,^35^,^36^,^37^,^38^,^39^,^40^,^41^. The characteristics of included studies are summarized in Table 1. Apart from studies not providing sufficient information for χ^2^ test, genotype distributions in controls were consistent with HWE in all studies for the polymorphism rs4986791 (*P* > 0.05, with a range of 0.3620 to 0.8560); while such distributions deviated from HWE expectation in two studies from the report by Tellería-Orriols *et al*.^20^ for the polymorphism rs4986790 (*P* = 0.0001), and perfectly fit in with the expectation in the other studies with *P* values ranging from 0.1690 to 0.9620.

### Meta-analysis results

**Figure 2:**
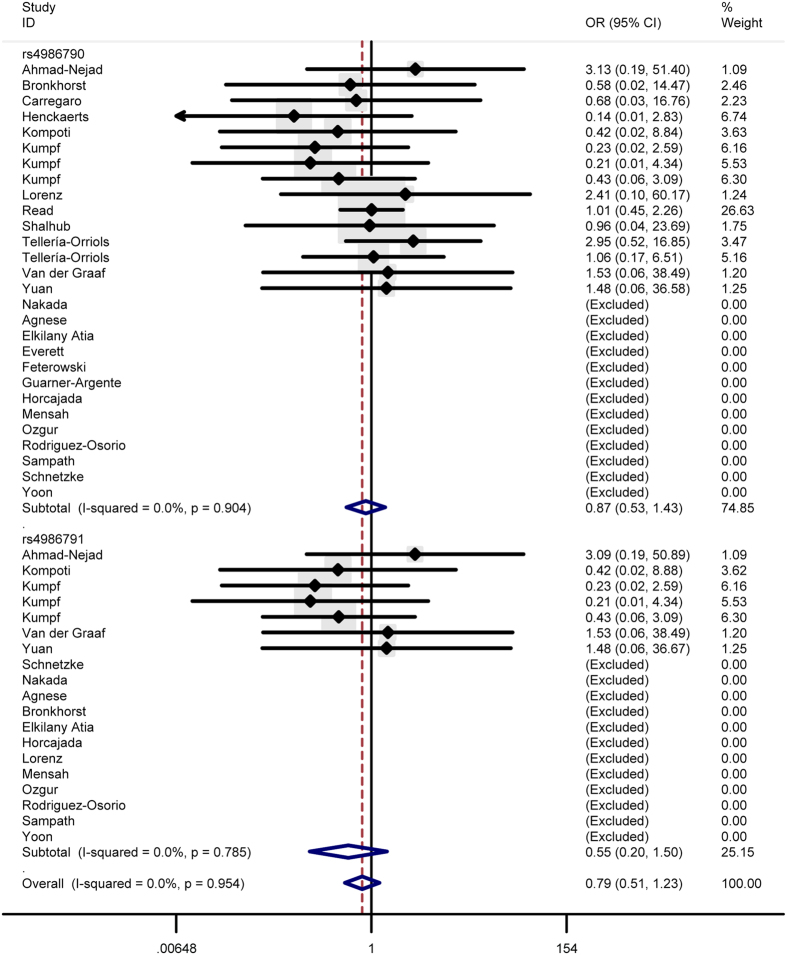
Forest plot of sepsis susceptibility associated with the polymorphisms rs4986790 and rs4986791 of toll like receptor 4 under the homozygous model.

**Figure 3:**
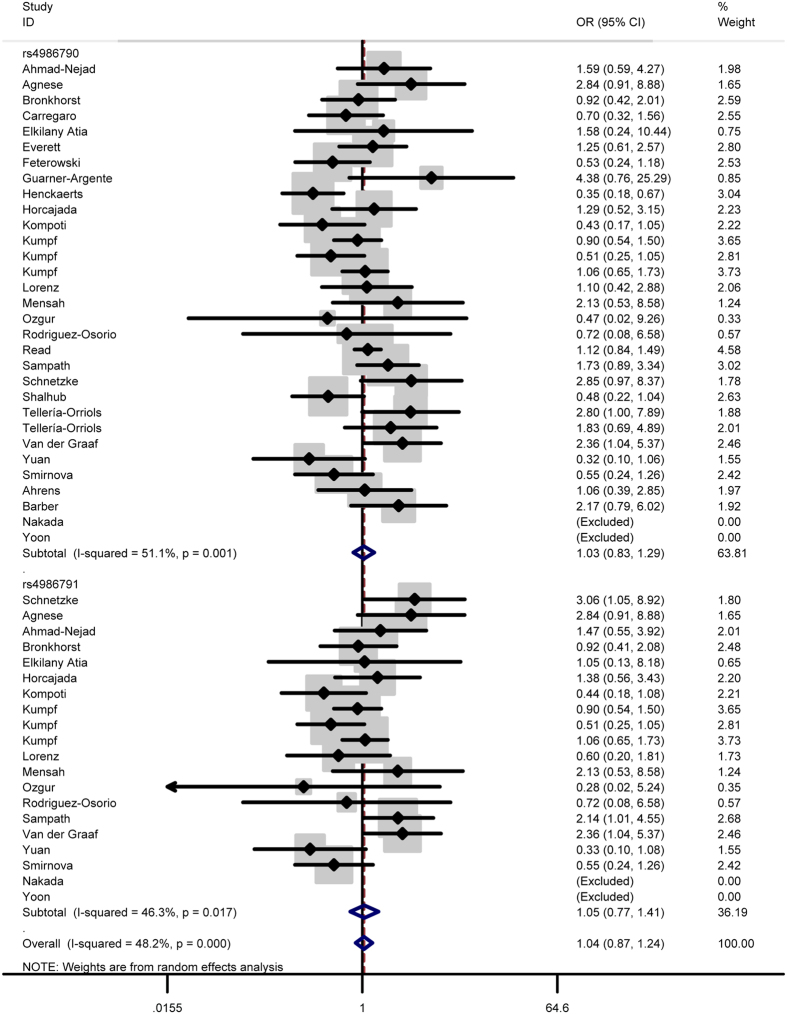
Forest plot of sepsis susceptibility associated with the polymorphisms rs4986790 and rs4986791 of toll like receptor 4 under the dominant model.

**Figure 4:**
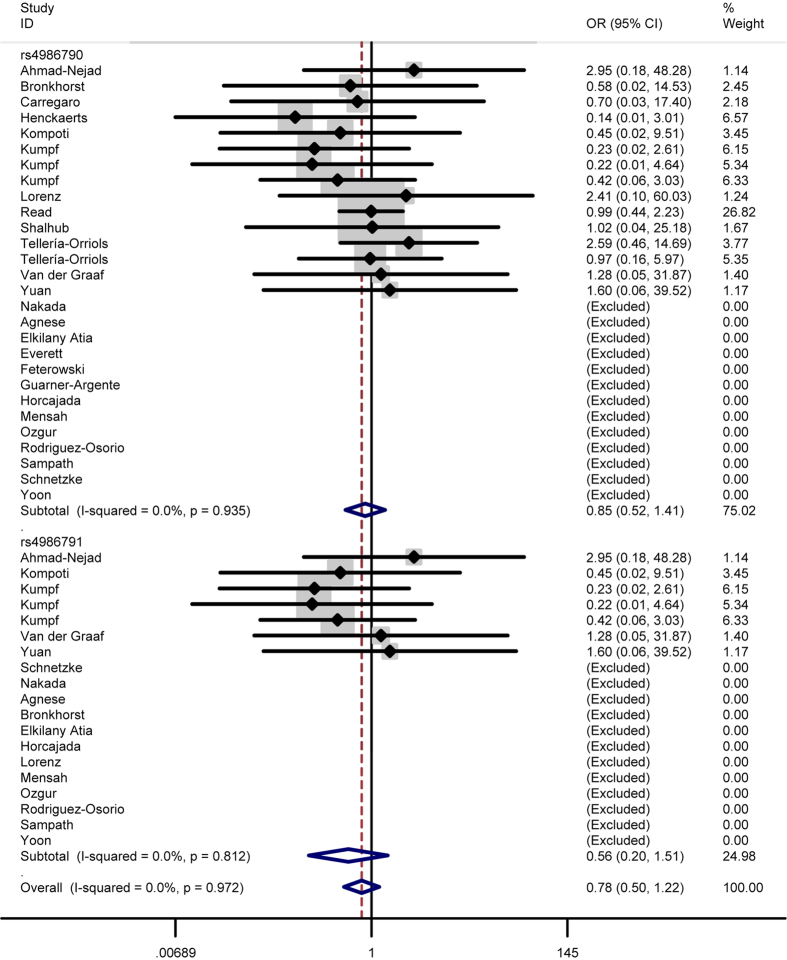
Forest plot of sepsis susceptibility associated with the polymorphisms rs4986790 and rs4986791 of toll like receptor 4 under the recessive model.

**Figure 5:**
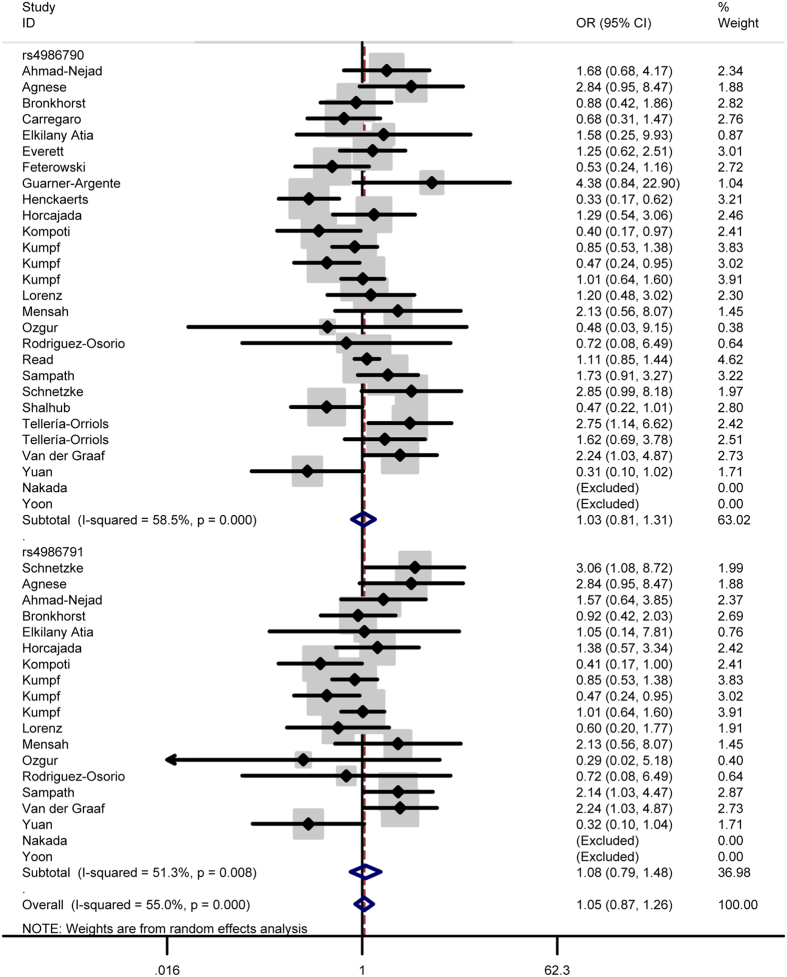
Forest plot of sepsis susceptibility associated with the polymorphisms rs4986790 and rs4986791 of toll like receptor 4 under the allele model.

**Figure 6:**
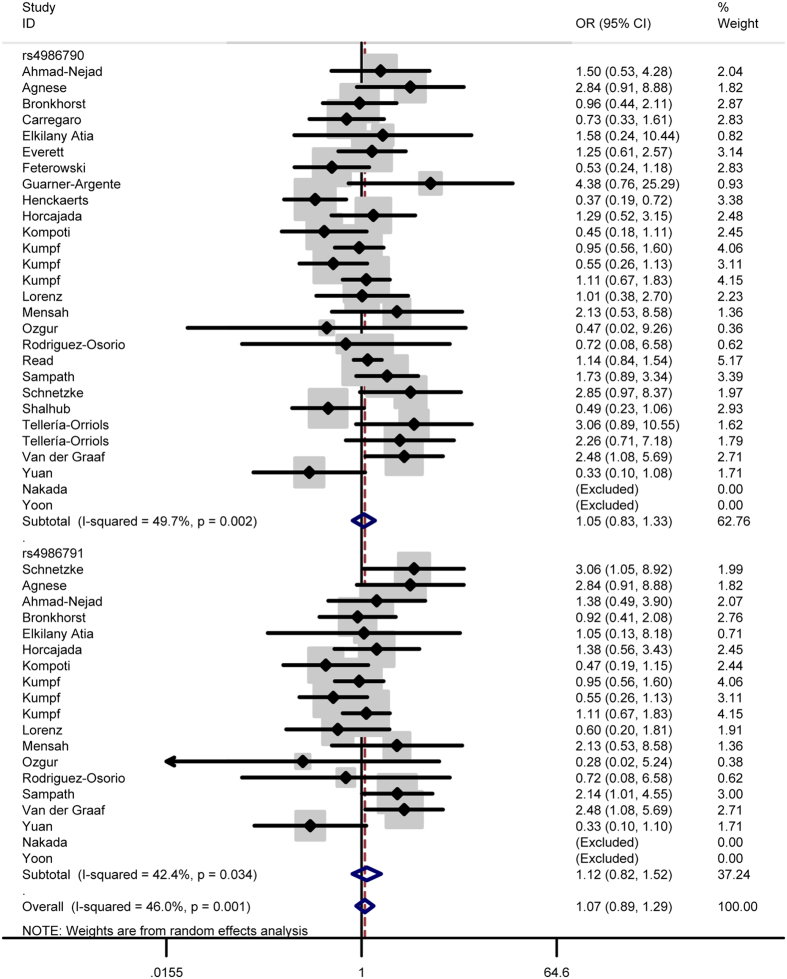
Forest plot of sepsis susceptibility associated with the polymorphisms rs4986790 and rs4986791 of toll like receptor 4 under the heterozygous model.

**Figure 7:**
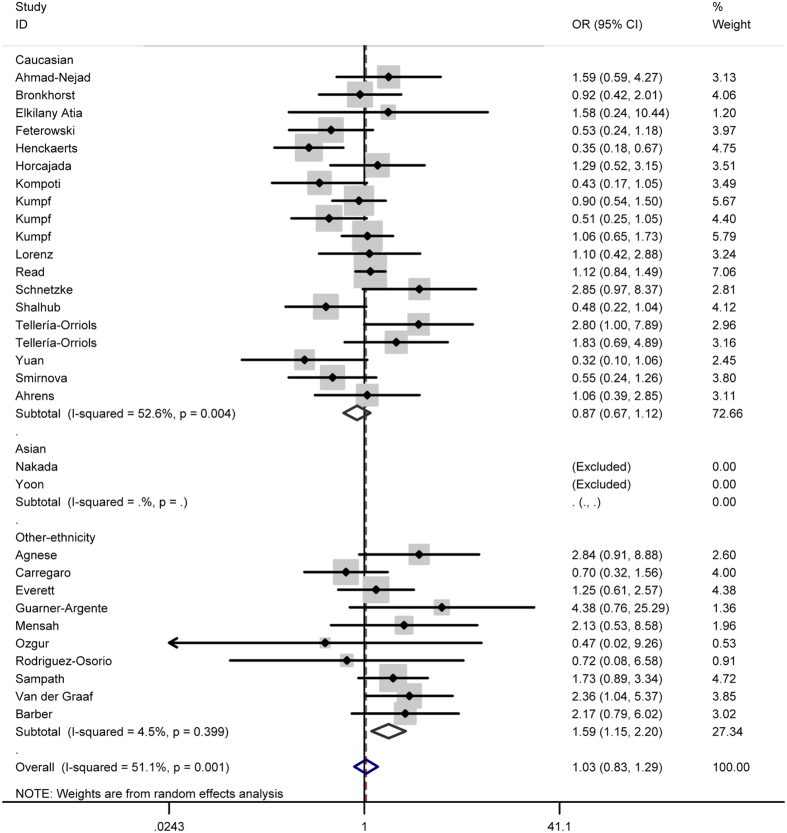
Forest plot of sepsis susceptibility associated with the polymorphism rs4986790 of toll like receptor 4 under GG + AG vs. AA model after stratification analysis by ethnicity.

In total analysis, the polymorphism rs4986790 of toll like receptor 4 gene showed no significant association with sepsis susceptibility under genetic models GG vs. AA (OR = 0.87, 95% CI = 0.53–1.43) (Fig. 2), GG + AG vs. AA (OR = 1.03, 95% CI = 0.83-1.29) (Fig. 3), GG vs. AA + AG (OR = 0.85, 95% CI = 0.52–1.41) (Fig. 4), allele G vs. allele A (OR = 1.03, 95% CI = 0.81–1.31) (Fig. 5) and AG vs. AA (OR = 1.05, 95% CI=0.83–1.33) (Fig. 6). Whereas, after stratification analysis by ethnicity, an increasing effect thereof on the sepsis risk was uncovered in other-ethnicity subgroup under GG + AG vs. AA (OR = 1.59 95% CI = 1.15–2.20) (Fig. 7), allele G vs. allele A (OR = 1.54 95% CI = 1.06–2.23) and AG vs. AA (OR = 1.5695% CI = 1.09–2.24) comparisons.

**Figure 8:**
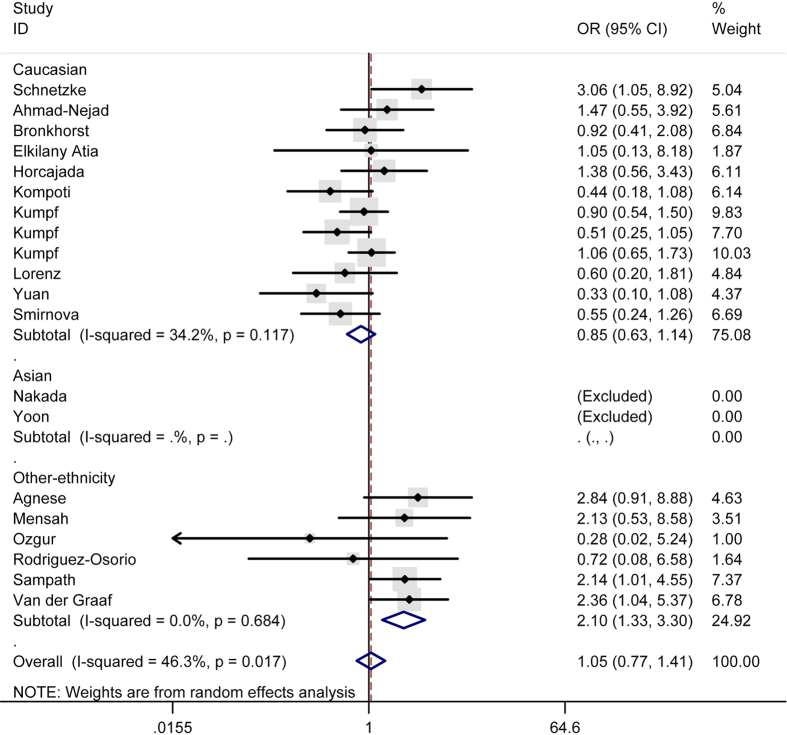
Forest plot of sepsis susceptibility associated with the polymorphism rs4986791 of toll like receptor 4 under the TT + CT vs. CC model after stratification analysis by ethnicity.

As for the polymorphism rs4986791, no significant influence thereof was detected in total analysis under the contrasts of TT vs. CC (Fig. 2), TT + CT vs. CC (Fig. 3), TT vs. CC + CT (Fig. 4), allele T vs. allele C (Fig. 5) and CT vs. CC (Fig. 6) (OR = 0.55, 95% CI = 0.20–1.50; OR = 1.05, 95% CI = 0.77–1.41; OR = 0.56, 95% CI = 0.20–1.51; OR = 1.08, 95% CI = 0.79–1.48; OR = 1.12, 95% CI = 0.82–1.52) either. However, this polymorphism significantly elevated the sepsis susceptibility in other-ethnicity subgroup under TT + CT vs. CC (Fig. 8), allele T vs. allele C and CT vs. CC models (OR = 2.10, 95% CI = 1.33–3.30; OR = 2.08, 95% CI = 1.34–3.22; OR = 2.13, 95% CI = 1.35–3.36) after stratified analysis by ethnicity.

In addition, we also examined possible effects of the combination between the polymorphisms rs4986790 and rs4986791 on the sepsis onset. As a result, the combination of these two polymorphism still lacked significant impact on the disease risk under homozygous model (OR = 0.79, 95% CI = 0.51–1.23) (Fig. 2), dominant model (OR = 1.04, 95% CI = 0.87–1.24) (Fig. 3), recessive model (OR = 0.78, 95% CI = 0.50–1.22) (Fig. 4), allele model (OR = 1.05, 95% CI = 0.87–1.26) (Fig. 5) and heterozygous model (OR = 1.07, 95% CI = 0.89–1.29) (Fig. 6).

### Test of heterogeneity

Significant heterogeneity was observed for the polymorphism rs4986790 under GG + GA vs. AA model, allele G vs. allele A model and GA vs. GG model (*P* = 0.001; *P* < 0.001; *P* = 0.002) as well as for the polymorphism rs4986791 under TT + CT vs. CC, allele T vs. allele C and CT vs. CC contrasts (*P* = 0.017; *P* = 0.008; *P* = 0.034), thus the random-effects model was applied to calculate pooled ORs in these cases. As for the other comparisons, the fixed-effects model was utilized considering the absence of significant heterogeneity (for rs4986790: *P* = 0.904 under GG vs. AA; *P* = 0.935 under GG + AG vs. AA; for rs4986791: *P* = 0.785 under TT vs. CC; *P* = 0.812 under TT + CT vs. CC).

As for the OR values for the effects of the combination between the two polymorphism, they were summarized applying the random-effects model under the dominant, allele and heterozygous models (*P* < 0.001; *P* < 0.001; *P* = 0.001) in view of the existence of significant heterogeneity, and pooled with the fixed-effects model under the homozygous and recessive models (*P* = 0.954; *P* = 0.972) because of the lack of significant heterogeneity.

### Sensitivity analysis and publication bias

**Figure 9:**
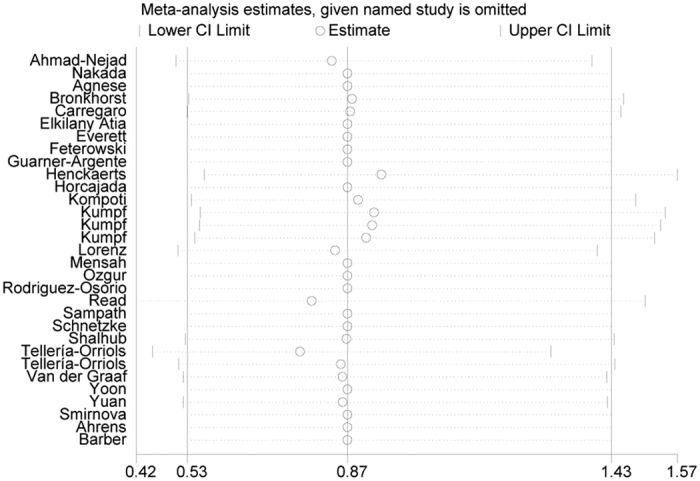
Sensitivity analysis for the polymorphism rs4986790.

**Figure 10:**
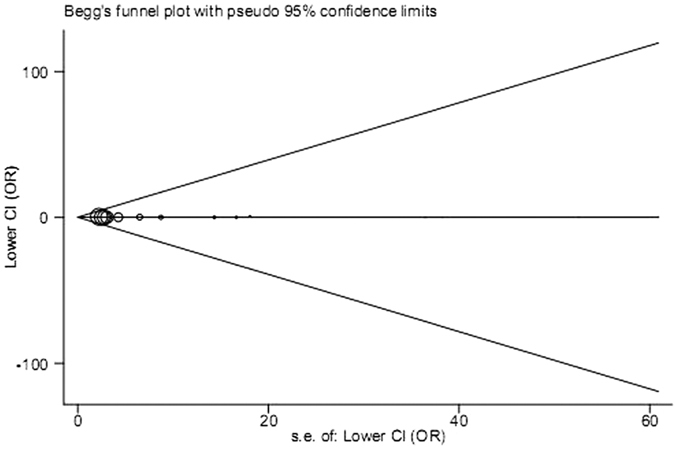
Begg’s funnel plot of publication bias examination on the studies for the polymorphism rs4986790 (model: GG vs. AA). Each point represents a separate study for the indicated association. Log(OR), natural logarithm of OR. Horizontal line, mean effect size.

We implemented sensitivity analysis to evaluate the stability of pooled results. We excluded each study one by one and observed that no pooled ORs was substantially affected (Fig. 9, with a range of lower CI from 0.42 to 0.56 and of upper CI from 1.30–1.57), indicating high stability of the meta-analysis results. Additionally, Begg’s funnel plot and Egger’s test were employed to evaluate publication bias between included studies. Consequently, all funnel plots seemed symmetrical (Fig. 10), implying no significant publication bias. What’s more, Egger’s test showed statistical evidence for these results (*P* = 0.674).

## Discussion

Sepsis is caused by infections and characterized by acute onset, rapid progression and high fatality rate, being a common postoperative complication of severe traumas and burns. SIRS may develop to septic stock and multiple organ dysfunction syndrome (MODS), and studies on SIRS, sepsis, severe sepsis, septic stock and MODS indicate significant differences in different individuals. Specifically, inflammatory responses may develop easily in some people, which is difficult to be controlled and then become MODS, while different results may occur in other people with the same SIRS. Moreover, people with similar SIRS or sepsis can show different prognosis when they adopt same therapeutic measures. All these evidence indicate that sepsis is affected by genetic factors as well. Therefore, it’s important to explore the roles of sepsis-related genes, which provides theoretical basis to understand sepsis pathogenesis.

TLRs, a group of main PRRs, can recognize pathogenic microorganisms through PAMPs, activate intracellular signal transduction pathways and induce the generation of innate immunity. Therefore, TLRs are involved in the pathogenic courses of numerous diseases and closely correlated with communicable diseases, tumors, cardiovascular diseases, autoimmune diseases and allergy. According to their positions in chromosome, genetic structures and amino acid sequences, 11 members of TLRs family are divided to 5 subfamilies. Among members in this family, toll like receptor 4, first found and reported by Medzhitov *et al*. in 1997^42^, is a transmembrane protein producing homologization to drosophila toll protein which is composed of extracellular region, transmembrane domain and intracellular region of leucine-rich-repeat (LRR), distributing mainly on the surface of cells (such as monocyte). This protein can recognize LPS of gram negative bacteria, mannan of fungus and soluble components of mycobacterium tuberculosis, and is the endogenous ligand of some heat shock proteins and fibronectins. Therefore, polymorphisms in coding gene for the protein may greatly affect systemic inflammation and immunoreaction.

For example, Chen *et al*. found that the polymorphism T-2242C in toll like receptor 4 gene might be related to higher sepsis morbidity rate and organ dysfunction^43^. Besides, Mansur *et al*. put forward in their study that the polymorphism rs11536889 in this gene was associated with renal, coagulation and hepatic organ failure in sepsis patients^44^. In addition, a study by Nachtigall *et al*. reported that the polymorphism rs4986790 might shorten the time-to-onset of severe sepsis or septic shock in patients permitted to intensive care units^45^. In the study by Child *et al*., the polymorphism rs4986790 was demonstrated to be involved in the severity of SIRS^46^. Additionally, this polymorphism was found to be correlated with septic shock induced by gram-negative bacteria^33^. However, there was research suggesting that such mutation might not influence the incidence of postoperative sepsis^16^. Such an inconclusive status was also true for another common polymorphism in the toll like receptor 4 gene, rs4976891. And these discrepancies might be attributed to but not limited to such aspects as various genetic backgrounds, different selection criteria for participants and uneven sample sizes.

Considering those discrepancies, we performed this meta-analysis to comprehensively analyze the associations of the rs4986790 and rs4986791 polymorphisms in toll like receptor 4 gene with sepsis susceptibility involving 51 relevant case-control studies. The results of this study showed that our studied polymorphisms, overall, had no significant association with the susceptibility of sepsis, but that both polymorphisms were related to increased risk of developing sepsis in other-ethnicity subgroups under corresponding genetic models after stratified analysis by ethnicity. Based on 6,537 sepsis patients and 8,832 controls, our findings had certain reliability, and sensitivity analysis also verified their stability. Compared to the previous individual case-control studies, our meta-analysis had a larger sample size and a more detailed stratification analysis by ethnicity, so our results were more reliable and comprehensive. In a recent meta-analysis about rs4986790 polymorphsism and sepsis susceptibility published by Zhu *et al*. in 2012^47^, 17 eligible articles were included, while our meta-analysis collected 28 related ones. Therefore, the present meta-analysis was an up-dated one with more eligible studies, suggesting our results could represent latest research results. However, there were some limitations in this meta-analysis, which might affect the accuracy of the result. For example, source limitation in literature searching might miss some relevant articles in other sources, thus leading to possible publication bias not detected even with Begg’s funnel plot or Egger’s test. In addition, possible effects from other relevant factors and gene-gene or gene-environment interactions were not incorporated in this study due to the insufficient data. Therefore, the results of the present meta-analysis should be applied with cautious.

In conclusion, the results of this meta-analysis showed that toll like receptor 4 gene rs4986790 and rs4986791 polymorphisms might not have independent association with sepsis susceptibility. In view of the advantages and disadvantages in this meta-analysis, the results should be further verified by better-designed studies based on larger sample size and more consideration of gene-gene and gene-environment interactions.

## Additional Information

**How to cite this article**: Liu, R. *et al*. The relationship between toll like receptor 4 gene rs4986790 and rs4986791 polymorphisms and sepsis susceptibility: A meta-analysis. *Sci. Rep.* **6**, 38947; doi: 10.1038/srep38947 (2016).

**Publisher’s note:** Springer Nature remains neutral with regard to jurisdictional claims in published maps and institutional affiliations.

## Figures and Tables

**Table 1:** Principal characteristics of the studies included in this meta-analysis.

SNP	First author-Year	Ethnicity	Control source	Genotyping method			Genotype and allele distribution										HWE
							Case					Control					
					Sample size		AA	AG	GG	A	G	AA	AG	GG	A	G	
**rs4986790**	Ahmad-Nejad-2011^17^	Caucasian	Hospital	PCR-RFLP	38	112	31	6	1	68	8	99	12	1	210	14	0.3640
	Nakada-2005^22^	Asian	Population	PCR-RFLP	86	214	86	0	0	172	0	214	0	0	428	0	/
	Agnese-2002^32^	Multi-ethnic	Population	PCR-RFLP	33	39	21	12	0	54	12	34	5	0	73	5	0.6690
	Bronkhorst-2013^27^	Caucasian	Hospital	PCR	79	139	68	11	0	147	11	118	20	1	256	22	0.8800
	Carregaro-2010^18^	Multi-ethnic	Population	PCR-RFLP	97	205	88	9	0	185	9	178	26	1	382	28	0.9620
	Elkilany Atia-2015^26^	Caucasian	Hospital	PCR	20	21	17	3	0	37	3	19	2	0	40	2	0.8190
	Everett-2007^38^	Undefined	Hospital	PCR	85	167	71	14	0	156	14	145	22	0	312	22	0.3620
	Feterowski-2003^16^	Caucasian	Hospital	PCR	153	154	143	10	0	296	10	135	19	0	289	19	0.4150
	Guarner-Argente-2010^35^	Undefined	Hospital	PCR-RFLP	6	105	4	2	0	10	2	97	8	0	202	8	0.6850
	Henckaerts-2009^40^	Caucasian	Population	PCR	407	293	393	14	0	800	14	264	27	2	555	31	0.1690
	Horcajada-2009^29^	Caucasian	Hospital	PCR	57	114	48	9	0	105	9	100	14	0	214	14	0.4850
	Kompoti-2015^25^	Caucasian	Hospital	PCR-RFLP	108	245	102	6	0	210	6	213	30	2	456	34	0.4170
	Kumpf-2010^41^	Caucasian	Population	PCR-RFLP	375	176	325	49	1	699	51	150	24	2	324	28	0.3620
	Kumpf-2010^41^	Caucasian	Population	PCR-RFLP	159	176	147	12	0	306	12	150	24	2	324	28	0.3620
	Kumpf-2010^41^	Caucasian	Population	PCR-RFLP	415	176	350	63	2	763	67	150	24	2	324	28	0.3620
	Lorenz-2002^33^	Caucasian	Population	PCR	91	73	80	10	1	170	12	65	8	0	138	8	0.6200
	Mensah-2009^23^	Multi-ethnic	Hospital	PCR	15	48	11	4	0	26	4	42	6	0	90	6	0.6440
	Ozgur-2009^30^	Undefined	Hospital	PCR	16	70	16	0	0	32	0	66	4	0	136	4	0.8060
	Rodriguez-Osorio-2013^28^	Mexican-Mestizo	Population	PCR-RFLP	44	126	43	1	0	87	1	122	4	0	248	4	0.8560
	Read-2001^31^	Caucasian	Population	TaqMan	1047	879	924	110	13	1958	136	787	81	11	1655	103	0.8250
	Sampath-2013^19^	Multi-ethnic	Hospital	multiplexed SBE	89	318	74	15	0	163	15	287	31	0	605	31	0.3610
	Schnetzke-2015^21^	Caucasian	Hospital	PCR	74	81	61	13	0	135	13	76	5	0	157	5	0.7740
	Shalhub-2009^14^	Caucasian	Hospital	PCR	147	451	139	8	0	286	8	400	50	1	850	52	0.6650
	Tellería-Orriols-2014^20^	Caucasian	Hospital	PCR	51	66	38	9	4	85	17	60	4	2	124	8	0.0001
	Tellería-Orriols-2014^20^	Caucasian	Hospital	PCR	102	66	85	14	3	184	20	60	4	2	124	8	0.0001
	Van der Graaf-2006^37^	Undefined	Population	PCR-RFLP	43	166	32	11	0	75	11	148	17	1	313	19	0.5120
	Yoon-2006^24^	Asian	Hospital	PCR-RFLP	154	179	154	0	0	308	0	179	0	0	358	0	/
	Yuan-2008^39^	Caucasian	Population	PCR-RFLP	85	409	82	3	0	167	3	364	44	1	772	46	0.7850
	Smirnova-2003^34^	Caucasian	Hospital	mutationseeker surveys	197	127	186	11		/	/	114	13		/	/	/
	Ahrens-2004^36^	Caucasian	Hospital	PCR-RFLP	50	306	45	5		/	/	277	29		/	/	/
	Barber-2004^13^	Multi-ethnic	Hospital	PCR	36	123	29	7		/	/	112	11		/	/	/
**rs4986791**							**CC**	**CT**	**TT**	**C**	**T**	**CC**	**CT**	**TT**	**C**	**T**	
	Schnetzke-2015^21^	Caucasian	Hospital	PCR	74	81	60	14	0	134	14	76	5	0	157	5	0.7740
	Nakada-2005^22^	Asian	Population	PCR-RFLP	86	214	86	0	0	172	0	214	0	0	428	0	/
	Agnese-2002^32^	Multi-ethnic	Population	PCR-RFLP	33	39	21	12	0	54	12	34	5	0	73	5	0.6690
	Ahmad-Nejad-2011^17^	Caucasian	Hospital	PCR-RFLP	38	112	31	6	1	68	8	98	13	1	209	15	0.4520
	Bronkhorst-2013^27^	Caucasian	Hospital	PCR	79	138	69	10	0	148	10	119	19	0	257	19	0.3850
	Elkilany Atia-2015^26^	Caucasian	Hospital	PCR	20	21	18	2	0	38	2	19	2	0	40	2	0.8190
	Horcajada-2009^29^	Caucasian	Hospital	PCR	57	114	48	9	0	105	9	101	13	0	215	13	0.5190
	Kompoti-2015^25^	Caucasian	Population	PCR-RFLP	108	245	102	6	0	210	6	214	29	2	457	33	0.3660
	Kumpf-2010^41^	Caucasian	Population	PCR	375	176	325	49	1	699	51	150	24	2	324	28	0.3620
	Kumpf-2010^41^	Caucasian	Population	PCR	159	176	147	12	0	306	12	150	24	2	324	28	0.3620
	Kumpf-2010^41^	Caucasian	Population	PCR	415	176	350	63	2	763	67	150	24	2	324	28	0.3620
	Lorenz-2002^33^	Caucasian	Population	PCR	91	73	85	6	0	176	6	65	8	0	138	8	0.6200
	Mensah-2009^23^	Multi-ethnic	Hospital	PCR	15	48	11	4	0	26	4	42	6	0	90	6	0.6440
	Ozgur-2009^30^	Undefined	Hospital	PCR	16	70	16	0	0	32	0	63	7	0	133	7	0.6600
	Rodriguez-Osorio-2013^28^	Mexican-Mestizo	Population	PCR	44	126	43	1	0	87	1	122	4	0	248	4	0.8560
	Sampath-2013^19^	Multi-ethnic	Hospital	multiplexed SBE	89	318	77	12	0	166	12	298	20	0	616	20	0.5630
	Van der Graaf-2006^37^	Undefined	Population	PCR	43	166	32	11	0	75	11	148	17	1	313	19	0.5120
	Yoon-2006^24^	Asian	Hospital	PCR-RFLP	154	179	154	0	0	308	0	179	0	0	358	0	/
	Yuan-2008^39^	Caucasian	Population	PCR	85	409	82	3	0	167	3	365	43	1	773	45	0.8210
	Smirnova-2003^34^	Caucasian	Hospital	mutationseeker surveys	197	127	186	11		/	/	114	13		/	/	/

Notes: HB, hospital-based; PCR, polymerase chain reaction; PCR-RFLP, PCR-restriction fragment length polymorphism; SBE, single-base extension; HWE, Hardy-Weinberg equilibrium.
